# Comprehensive analysis of DTYMK for estimating the immune microenvironment, diagnosis, prognosis effect in patients with lung adenocarcinoma

**DOI:** 10.18632/aging.204308

**Published:** 2022-09-27

**Authors:** Xi Chen, Yixiao Yuan, Fan Zhou, Xiaobin Huang, Jun Pu, Xiaoqun Niu, Xiulin Jiang, Xiaoyan Ding

**Affiliations:** 1 Department of Neurosurgery, The Second Affiliated Hospital of Kunming Medical University, Kunming 650223, Yunnan Province, China; 2 Key Laboratory of Molecular Oncology and Epigenetics, The First Affiliated Hospital of Chongqing Medical University, Chongqing 400016, China; 3 Department of Hematology, The Second Affiliated Hospital of Kunming Medical University, Kunming 650223, Yunnan Province, China; 4 Department of Respiratory Medicine, The Second Affiliated Hospital of Kunming Medical University, Kunming 650223, Yunnan Province, China; 5 College of Life Science, University of Chinese Academy of Sciences, Beijing 100049, China; 6 Department of Oncology, The People’s Hospital of Lishui, Lishui 323000, Zhejiang, China

**Keywords:** DTYMK, lung adenocarcinoma, prognosis biomarker, cell proliferation, cell cycle

## Abstract

The expression of deoxythymidylate kinase (DTYMK) is up-regulated in liver cancer. However, the underlying biological function and potential mechanisms of DTYMK driving the progression of lung adenocarcinoma remains unclear. In this study, we investigated the role of DTYMK in lung adenocarcinoma and found that the expression of DTYMK in LUAD tissues was significantly higher than that of DTYMK expression in adjacent normal tissues. Kaplan-Meier survival analysis showed that patients with higher DTYMK expression correlated with adverse prognosis. ROC curve analysis showed that the AUC value of DTYMK was 0.914. Correlation analysis showed that DTYMK expression was associated with immune infiltration in LUAD. Finally, we determine that DTYMK regulated cell proliferation, cell migration, and cell cycle of lung adenocarcinoma *in vitro*. In conclusion, our data demonstrated that DTYMK was correlated with progression and immune infiltration, and could serve as a prognostic biomarker for lung adenocarcinoma.

## INTRODUCTION

Lung cancer is the most lethal tumor of respiratory system [1]. Lung adenocarcinomas are found in nearly 40% of lung cancers, and squamous cell carcinomas and large cell carcinomas are the other two subtypes of NSCLC [2]. For patients with lung adenocarcinoma (LUAD), the overall survival times is less than 20% [2]. It is very important to study the initiation and metastasis of lung cancer and identify new biomarkers for the prevention and diagnosis of lung cancer.

DTYMK, a nuclear deoxythymidylate kinase, plays an important role in catalyzes the process of deoxy-TMP phosphorylation. Relevant studies have reported that DTYMK exerts a pivotal effect on multiple physiological processes including genome integrity and neuronal survival [3]. It has been reported that DTYMK was dramatically upregulated in HCC and correlated with worse overall survival [4]. However, the potential biological and clinical value of DTYMK in lung cancer remains unclear.

In this finding, we comprehensively analysis the expression patterns, clinical relevance and prognostic value of DTYMK in lung cancer. Finally, the biological event of DTYMK in lung cancer progression was examined by loss of function. Our findings may provide insight into the mechanism and role of DTYMK in LUAD.

## MATERIALS AND METHODS

### Data processing

The normalized gene expression matrix and clinical information data in various tumor and normal tissue samples was downloaded from TCGA database and GenotypeTissue Expression (GTEx) databases.

### Gene enrichment analysis based on DTYMK co-expressed genes

We examined the co-expression genes of DTYMK by using LinkedOmics (http://www.linkedomics.org/login.php) [5]. We also performed the GO and KEGG enrichment analysis of DTYMK in LUAD by using the R package “clusterProfiler”.

### Diagnostic and prognosis values

In this manuscript, we mainly used the ROC curve and Kaplan-Meier Plotter to examine the potential diagnostic and prognosis values of DTYMK in LUAD [6].

### Immune infiltration analysis of DTYMK in LUAD

The TIMER database is a bioinformatics web tool for comprehensively analyzing tumor-infiltrating immune cells (TIICs) [7]. The correlation between DTYMK and various human immune cells in LUAD was analyzed in TIMER.

### Loss of function assay

We conducted the CCK8 assay and transwell assay to examine the potential biological function of DTYMK on the cell behavior of LUAD. Above assay mainly refer to previous and published literature [8].

### Statistical analyses

The statistical for Figure analyses were performed using R (V 3.6.3), and ROC curves were used to detect DTYMK cutoff values using pROC packages. P < 0.05 (*), P < 0.01 (**) and P < 0.001 (***), were significant.

## RESULTS

### DTYMK was over-expression in pan-cancer

**Figure 1:**
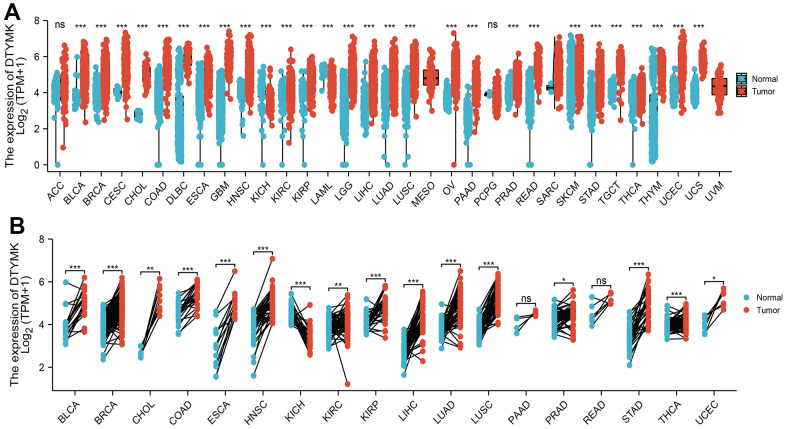
**Expression pattern of DTYMK from the perspective of pan-cancer.** (**A**) DTYMK was highly expressed in 24 of the 33 cancers compared with normal tissue. (**B**) Relative DTYMK expression in paired lung cancer and noncancerous tissues examined by TCGA databases. ACC: Adrenocortical carcinoma, BLCA: Bladder Urothelial Carcinoma, BRCA: Breast invasive carcinoma, CESC: Cervical squamous cell carcinoma and endocervical adenocarcinoma, CHOL: Cholangiocarcinoma, COAD: Colon adenocarcinoma, DLBC: Lymphoid Neoplasm Diffuse Large B-cell Lymphoma, ESCA: Esophageal carcinoma, GBM: Glioblastoma multiforme, HNSC: Head and Neck squamous cell carcinoma, KICH: Kidney: Chromophobe, KIRC: Kidney renal clear cell carcinoma, KIRP: Kidney renal papillary cell carcinoma, LAML: Acute Myeloid Leukemia, LUAD: Brain Lower Grade Glioma, LIHC: Liver hepatocellular carcinoma, LUAD: Lung adenocarcinoma, LUSC: Lung squamous cell carcinoma, MESO: Mesothelioma, OV: Ovarian serous cystadenocarcinoma, PAAD: Pancreatic adenocarcinoma, PCPG: Pheochromocytoma and Paraganglioma, PRAD: Prostate adenocarcinoma, READ: Rectum adenocarcinoma, SARC: Sarcoma, SKCM: Skin Cutaneous Melanoma, STAD: Stomach adenocarcinoma, TGCT: Testicular Germ Cell Tumors, THCA: Thyroid carcinoma, THYM: Thymoma, UCEC: Uterine Corpus Endometrial Carcinoma, UCS: Uterine Carcinosarcoma, UVM: Uveal Melanoma. NS: P >0.05,*P < 0.05, **P < 0.01, ***P < 0.001.

**Figure 2:**
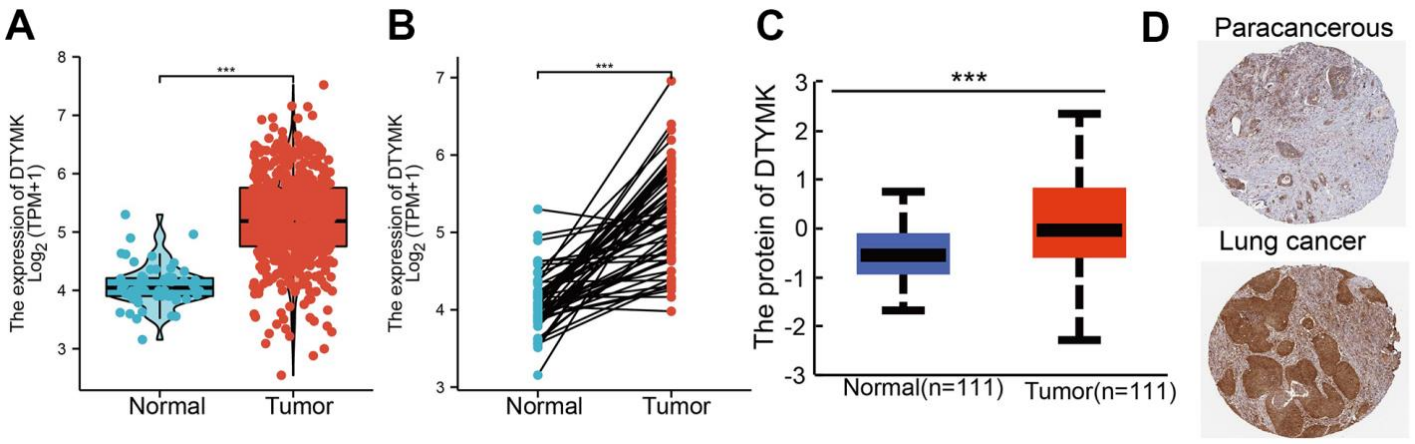
**DTYMK RNA and protein expression in LUAD.** (**A**) DTYMK mRNA expression levels in 535 lung cancer patients and matched adjacent normal samples. (**B**) Relative DTYMK expression in 54 paired lung cancer and noncancerous tissues. (**C**) DTYMK protein expression level based on CPTAC. (**D**) DTYMK protein levels based on Human Protein Atlas. ***P < 0.001.

We used TCGA and GTEx datasets to examine the levels of DTYMK in various cancer types. Results confirmed that DTYMK was up-regulated in many human cancer (Figure 1A, 1B). We also found that DTYMK expression was significantly increased compared with normal tissues in both unpaired and paired expression (Figure 2A, 2B). In addition, we showed that DTYMK protein level was elevated in LUAD than in normal tissue (Figure 2C, 2D).

### DTYMK was related to the clinical parameters in LUAD

**Figure 3:**
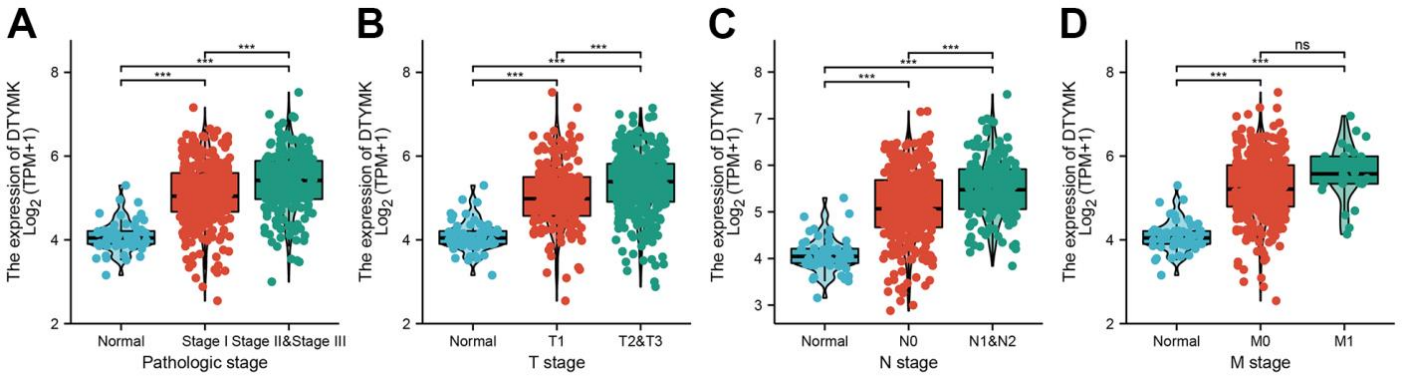
**Clinical significance of DTYMK in lung adenocarcinoma.** Correlation between DTYMK expression and clinical parameters, including (**A**) pathological stage, (**B**–**D**) TNM stage NS: P >0.05, ***P < 0.001.

**Figure 4:**
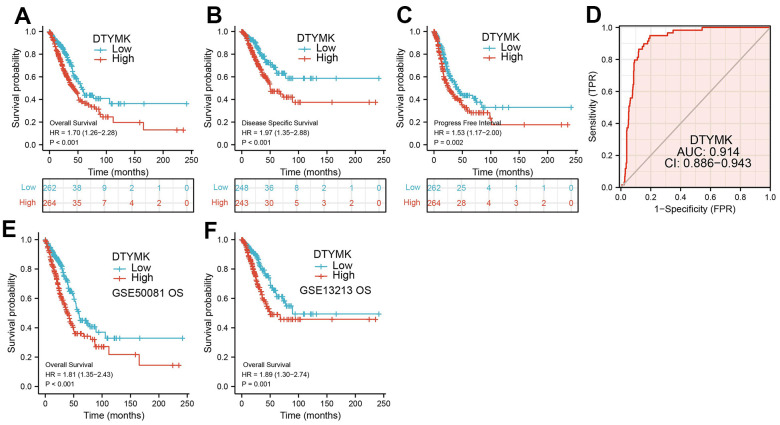
**ROC and Kaplan-Meier curves of DTYMK.** (**A**–**C**) Kaplan–Meier survival curves showed that lung adenocarcinoma patients with high DTYMK expression exhibited poor overall survival, disease-specific survival and progression-free survival of DTYMK in LUAD determine by TCGA-LUAD dataset. (**D**) ROC curves were used to determine the diagnostic value of DTYMK in lung adenocarcinoma. (**E**, **F**) Kaplan–Meier survival curves showed that lung adenocarcinoma patients with high DTYMK expression exhibited poor overall survival determine by GEO dataset.

**Figure 5:**
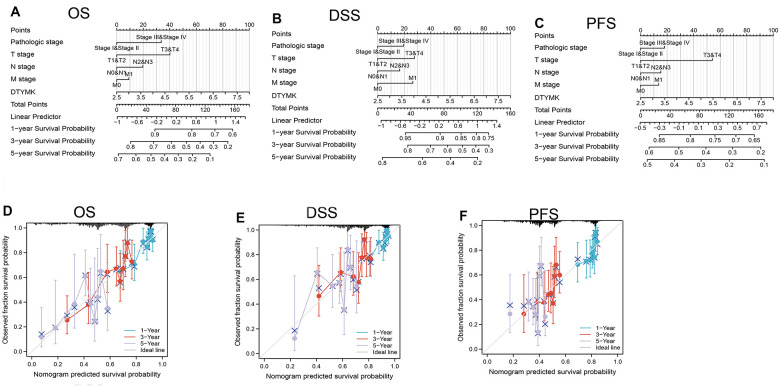
**Construction and performance validation of the DTYMK based nomogram for lung adenocarcinoma patients.** Nomogram to predict the (**A**) overall survival, (**B**) disease-specific survival, and (**C**) progression-free survival for lung cancer patients. The calibration curve and Hosmer–Lemeshow test of nomograms in the TCGA- lung adenocarcinoma cohort for (**D**) overall survival, (**E**) disease-specific survival and (**F**) progression-free survival.

The correlations between DTYMK expression and patients’ clinicopathological parameters, including TNM stage and age was analyzed using the TCGA-LUAD. Results show that up-regulation of DTYMK was related to the adverse pathological stage, and TNM stage (Table 1 and Figure 3A–3D). We also found that patients with higher DTYMK expression had worse OS, DSS, and PFS in patients with LUAD (Figure 4A–4C). ROC curve analysis results suggested that the AUC value of DTYMK is 0.914, 95% CI = 0.886-0.943(Figure 4D). Above results was validation by GEO datasets (Figure 4E, 4F). In the univariate Cox regression analysis models, pathologic stage, and TNM stage, and DTYMK were closely associated with OS in LUAD. In the multivariate models of Cox regression analysis, DTYMK closely associated with OS in LUAD patients, along with pathological stage and T stage (Table 2). We established a nomogram and showed that this model could be predicted the overall survival of patients with LUAD (Figure 5A–5F).

**Table 1:** Clinical characteristics of the LUAD patients.

**Characteristic**	**Low expression of DTYMK**	**High expression of DTYMK**	**p**
N	267	268	
T stage, n (%)			< 0.001
T1	113 (21.2%)	62 (11.7%)	
T2	121 (22.7%)	168 (31.6%)	
T3	22 (4.1%)	27 (5.1%)	
T4	9 (1.7%)	10 (1.9%)	
N stage, n (%)			< 0.001
N0	197 (38%)	151 (29.1%)	
N1	33 (6.4%)	62 (11.9%)	
N2	24 (4.6%)	50 (9.6%)	
N3	0 (0%)	2 (0.4%)	
M stage, n (%)			0.003
M0	176 (45.6%)	185 (47.9%)	
M1	4 (1%)	21 (5.4%)	
Pathologic stage, n (%)			< 0.001
Stage I	174 (33%)	120 (22.8%)	
Stage II	53 (10.1%)	70 (13.3%)	
Stage III	30 (5.7%)	54 (10.2%)	
Stage IV	5 (0.9%)	21 (4%)	
Age, median (IQR)	67 (60, 74)	64 (58, 71)	0.003

**Table 2:** Univariate and multivariate Cox regression analyses of different parameters on overall survival in lung adenocarcinoma.

**Characteristics**	**Total(N)**	**Univariate analysis**			**Multivariate analysis**	
		**Hazard ratio (95% CI)**	**P value**		**Hazard ratio (95% CI)**	**P value**
Pathologic stage	518					
Stage I&Stage II	411					
Stage III&Stage IV	107	2.664 (1.960-3.621)	<0.001		5.497 (1.987-15.205)	0.001
T stage	504					
T1	175					
T2&T3	329	1.658 (1.175-2.341)	0.004		1.690 (1.077-2.649)	0.022
N stage	510					
N0&N1	437					
N2&N3	73	2.321 (1.631-3.303)	<0.001		0.485 (0.173-1.358)	0.169
M stage	377					
M0	352					
M1	25	2.136 (1.248-3.653)	0.006		0.353 (0.114-1.095)	0.072
Smoker	512					
No	72					
Yes	440	0.894 (0.592-1.348)	0.591			
DTYMK	526	1.453 (1.199-1.762)	<0.001		1.203 (0.938-1.543)	0.014

### PPI network and functional annotation

**Figure 6:**
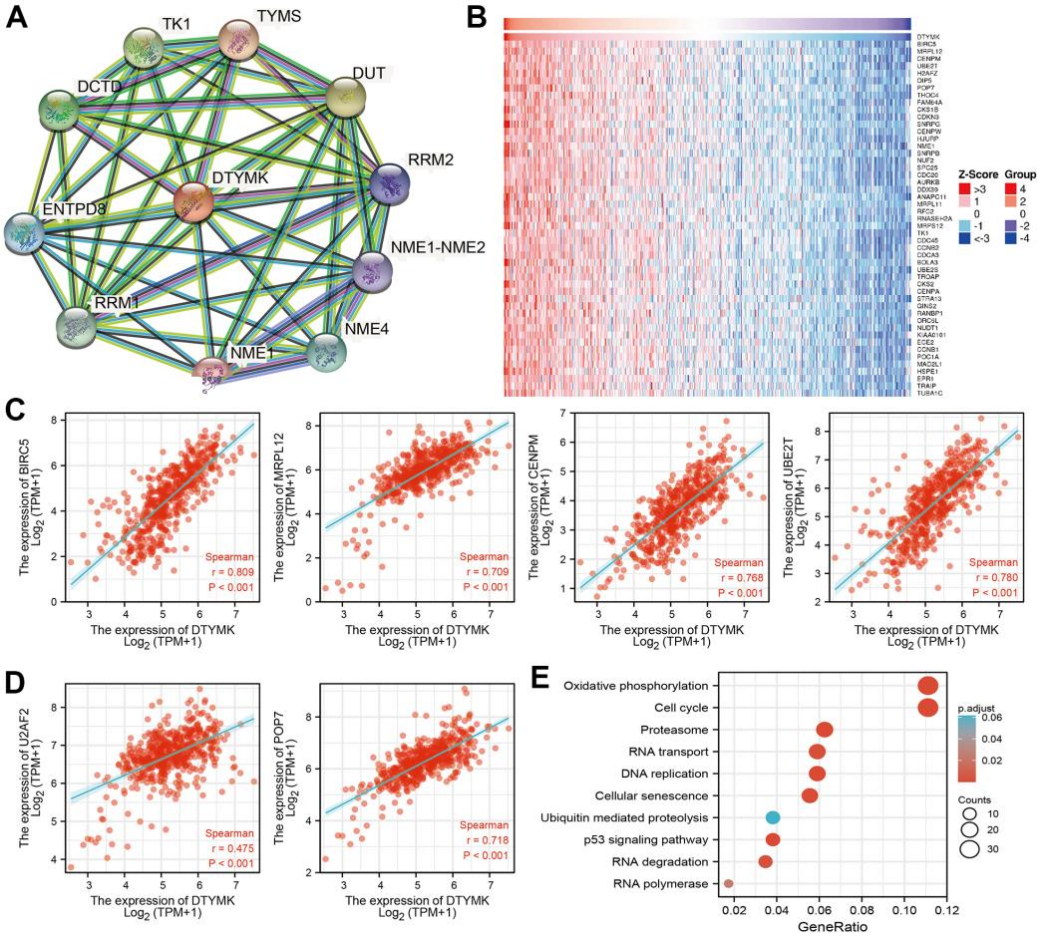
**PPI network and functional enrichment analysis.** (**A**) The protein-protein network of DTYMK examine by STRING database. (**B**–**D**) The correlation analysis of DTYMK expression and its top 100 co-expressed gene network. (**E**) Functional enrichment analysis of co-expressed genes.

**Figure 7:**
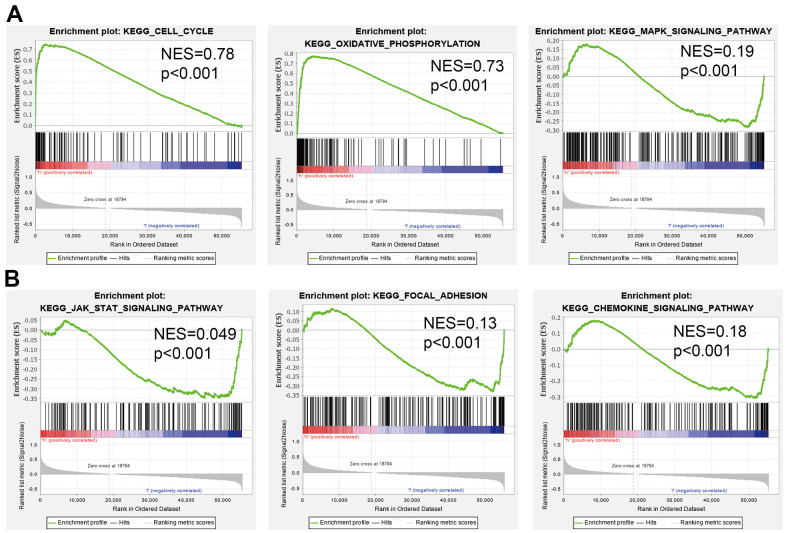
**Identification of DTYMK related signaling pathways in lung adenocarcinoma.** (**A**, **B**) Identification of DTYMK related signaling pathways by GSEA software.

As shown in Figure 6A, STRING database utilized to construct the PPI network. Furthermore, we also obtained the top co-expressed genes (Figure 6B–6D). Functional analysis showed that DTYMK mainly related to Oxidative phosphorylation, Cell cycle, Proteasome, 53 signaling pathway, RNA degradation, and RNA polymerase (Figure 6E). GSEA result show that DTYMK mainly involved in cell cycle, Oxidative phosphorylation, Focal adhesion, and chemokine signaling pathway (Figure 7A, 7B).

### Immune infiltration analysis of DTYMK

**Figure 8:**
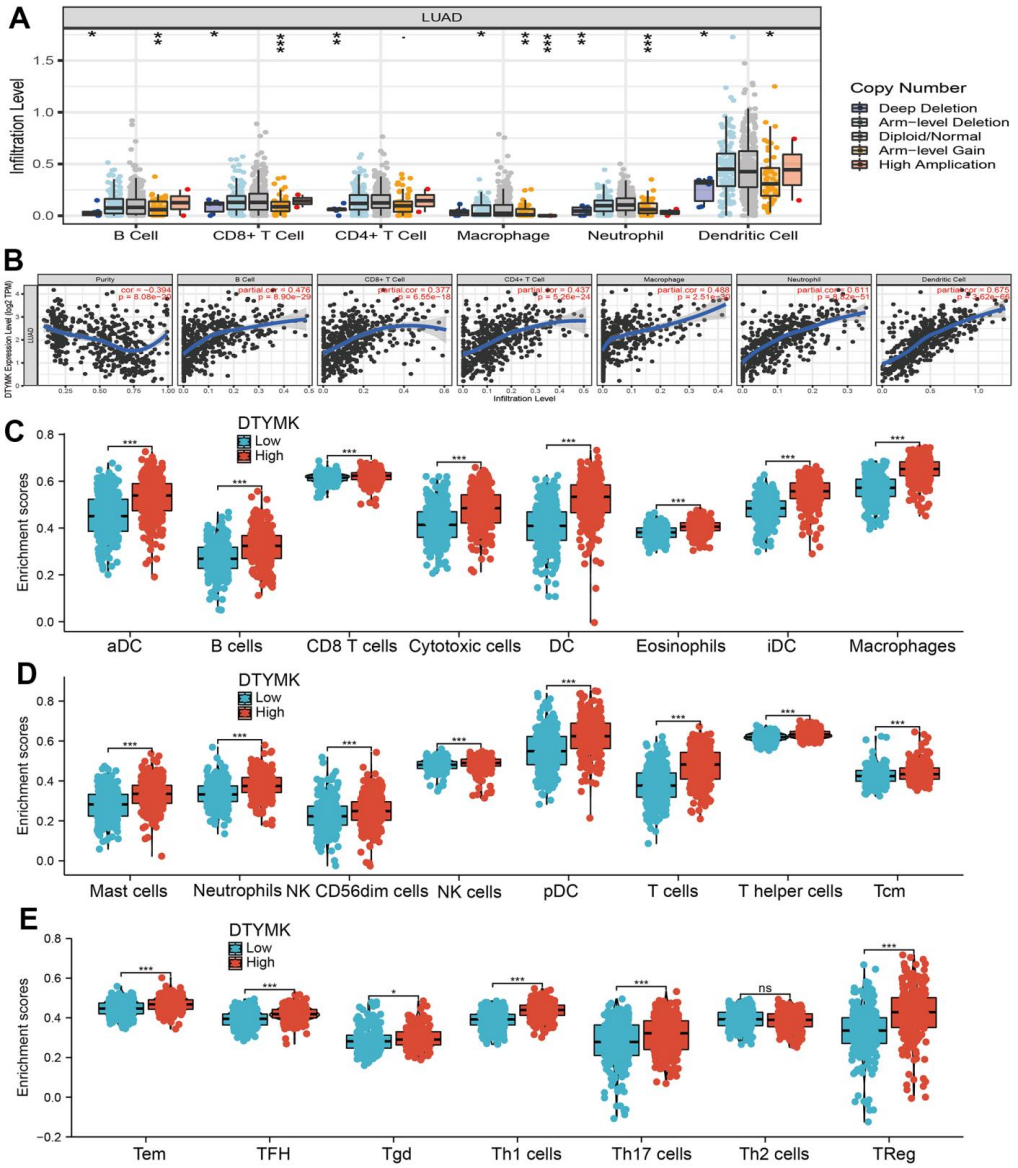
**Correlation analysis of DTYMK expression and infiltration levels of immune cells in LUAD.** (**A**) The correlation between DTYMK expression and somatic copy number alterations examine by TIMER. (**B**) The correlation between DTYMK expression and the infiltration levels of B cells, CD4+ T cells, CD8+ T cells, dendritic cells, Macrophages and Neutrophils. (**C**–**E**) Box plots of the correlations between DTYMK or molecular model expression and infiltration levels of immune cells. NS: P >0.05,*P < 0.05, **P < 0.01, ***P < 0.001.

Firstly, the TIMER database was employed to evaluate the relationship between DTYMK levels and the level of TIICs infiltration in TCGA-LUAD. Different infiltration levels of TIICs appeared to be related to the alteration of DTYMK, including various immune cells (Figure 8A). Moreover, we show that *DTYMK* was significantly correlated with the level of diverse human immune cells (Figure 8B). Finally, we determined that elevated *DTYMK* expression was positive related to the abundance of 22 human immune cells (Figure 8C–8E).

### Knockdown of *DTYMK* inhibited LUAD progression

**Figure 9:**
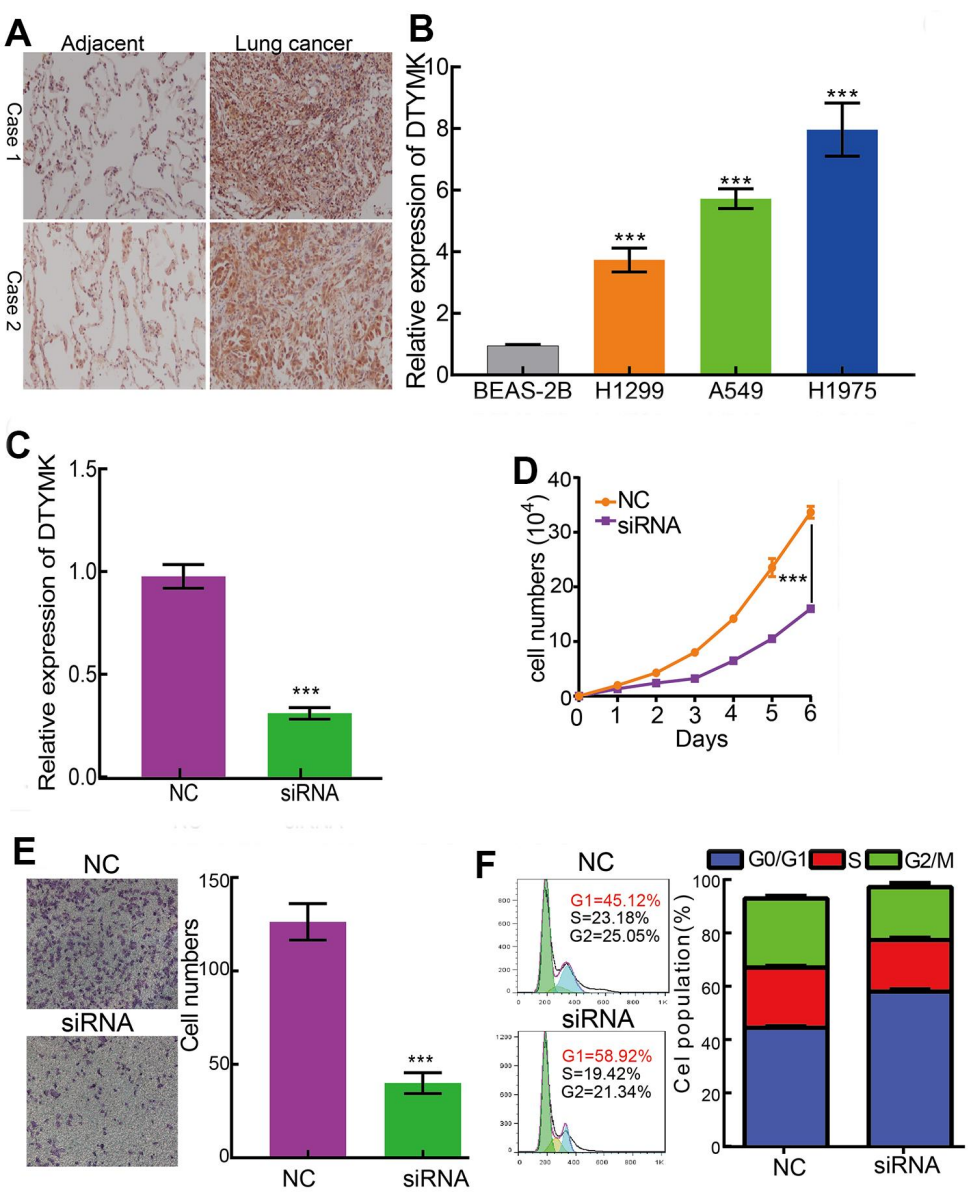
**DTYMK regulates LUAD cell proliferation, migration and cell cycle.** (**A**) IHC assay detect the protein of DTYMK in lung adenocarcinoma cancerous. (**B**) qPCR assay examine the expression level of DTYMK in lung adenocarcinoma cancerous cell lines, including A549, H1299, and H1975, compared to normal human bronchial epithelial cell line: BEAS-2B. (**C**) Establishment of DTYMK knockdown cell lines in H1975 verified by Real-time RT-PCR (**D**, **E**) Knockdown of DTYMK significantly inhibits cell proliferation and migration in H1975 cells, as measured by CCK8 and transwell assays. (**F**) Depletion of DTYMK increased G0/G1 phase arrested cells. NC=negative control, siRNA= DTYMK siRNA ***p < 0.001.

We showed that *DTYMK* was up-regulated in A549 and H1975 cells (Figure 9A, 9B). Loss of function assays suggested that knockdown of DTYMK inhibited the cell growth and migration abilities of LUAD cells (Figure 9C–9E). Furthermore, *DTYMK* knock-down led to increased G0/G1 phase arrested cells (Figure 9F).

## DISCUSSION

It has been confirmed that DTYMK mainly catalyzes the last reaction of the deoxyribonucleoside triphosphate (dTTP) production pathways [9]. According to previous studies, we showed that DTYMK was significantly different in LUAD and normal tissues and associated with OS in LUAD patients.

In this finding, we evaluated the expression of DTYMK in LUAD dependent databases such as TCGA, GTEx, and UALCAN. We found that DTYMK was up-regulated in LUAD. Moreover, DTYMK expression was increased in the different cancer stages of LUAD, compared with the normal control group. Next, to examine whether DTYMK can be used as a prognostic biomarker, we analyzed the relationship between DTYMK in LUAD and OS, DSS, and PFI and plotted the survival curve using Kaplan-Meier plotter databases. Notably, increased DTYMK expression was associated with worse OS, DSS, and PFS. Above results showed that up-regulation of DTYMK was associated with a worse prognosis in LUAD patients.

Previous studies reported that DTYMK was significant for neuronal survival [3]. In this finding, we show that DTYMK are involved in Oxidative phosphorylation, Proteasome, 53 signaling pathway, RNA degradation, and RNA polymerase.

Our study showed that DTYMK is closely correlated with immune cells, suggesting that DTYMK plays a critical role in lung cancer immunity. Immune checkpoints are pairs of receptor-ligand molecules that interact to suppress immune responses, and tumors can protect themselves by using immune checkpoints to evade immune clearance [10]. Our study also found that DTYMK is associated with immune in human lung cancer types. In conclusion, DTYMK may affect the prognosis of various cancers and is related to immune infiltration.

We have analyzed the role of DTYMK in LUAD and the potential clinical value of DTYMK in lung cancer. However, the current study still had some limitations. First, there were no *in vivo* experiments to verify the role of DTYMK in LUAD. Overall, our results confirmed that DTYMK may be a potential novel prognostic biomarker for LUAD, which may improve our understanding of the translational use of DTYMK in LUAD prognosis and therapy.
